# Functional and Molecular Properties of DYT-SGCE Myoclonus-Dystonia Patient-Derived Striatal Medium Spiny Neurons

**DOI:** 10.3390/ijms22073565

**Published:** 2021-03-30

**Authors:** Anna Kutschenko, Selma Staege, Karen Grütz, Hannes Glaß, Norman Kalmbach, Thomas Gschwendtberger, Lisa M. Henkel, Johanne Heine, Anne Grünewald, Andreas Hermann, Philip Seibler, Florian Wegner

**Affiliations:** 1 Department of Neurology, Hannover Medical School, Carl-Neuberg-Str. 1, 30625 Hannover, Germany; kutschenko.anna@mh-hannover.de (A.K.); staege.selma@mh-hannover.de (S.S.); norman.kalmbach@googlemail.com (N.K.); gschwendtberger.thomas@mh-hannover.de (T.G.); henkel.lisa@mh-hannover.de (L.M.H.); heine.johanne@mh-hannover.de (J.H.); 2 Center for Systems Neuroscience, Bünteweg 2, 30559 Hannover, Germany; 3 Institute of Neurogenetics, University of Lübeck, Ratzeburger Allee 160, 23538 Lübeck, Germany; karen.gruetz@neuro.uni-luebeck.de (K.G.); anne.gruenewald@neuro.uni-luebeck.de (A.G.); philip.seibler@neuro.uni-luebeck.de (P.S.); 4 Translational Neurodegeneration Section “Albrecht-Kossel“, Department of Neurology, University Medical Center, University of Rostock, Gehlsheimer Str. 20, 18147 Rostock, Germany; hannes.glass@med.uni-rostock.de (H.G.); andreas.hermann@med.uni-rostock.de (A.H.); 5 German Center for Neurodegenerative Diseases Rostock/Greifswald, 18147 Rostock, Germany; 6 Center for Transdisciplinary Neurosciences Rostock (CTNR), University Medical Center, University of Rostock, 18147 Rostock, Germany

**Keywords:** DYT-SGCE, myoclonus-dystonia, induced pluripotent stem cells, striatal medium spiny neurons, calcium dynamics, patch-clamp electrophysiology, GABAergic synaptic density

## Abstract

Myoclonus-dystonia (DYT-SGCE, formerly DYT11) is characterized by alcohol-sensitive, myoclonic-like appearance of fast dystonic movements. It is caused by mutations in the *SGCE* gene encoding ε-sarcoglycan leading to a dysfunction of this transmembrane protein, alterations in the cerebello-thalamic pathway and impaired striatal plasticity. To elucidate underlying pathogenic mechanisms, we investigated induced pluripotent stem cell (iPSC)-derived striatal medium spiny neurons (MSNs) from two myoclonus-dystonia patients carrying a heterozygous mutation in the *SGCE* gene (c.298T>G and c.304C>T with protein changes W100G and R102X) in comparison to two matched healthy control lines. Calcium imaging showed significantly elevated basal intracellular Ca^2+^ content and lower frequency of spontaneous Ca^2+^ signals in SGCE MSNs. Blocking of voltage-gated Ca^2+^ channels by verapamil was less efficient in suppressing KCl-induced Ca^2+^ peaks of SGCE MSNs. Ca^2+^ amplitudes upon glycine and acetylcholine applications were increased in SGCE MSNs, but not after GABA or glutamate applications. Expression of voltage-gated Ca^2+^ channels and most ionotropic receptor subunits was not altered. SGCE MSNs showed significantly reduced GABAergic synaptic density. Whole-cell patch-clamp recordings displayed elevated amplitudes of miniature postsynaptic currents and action potentials in SGCE MSNs. Our data contribute to a better understanding of the pathophysiology and the development of novel therapeutic strategies for myoclonus-dystonia.

## 1. Introduction

Myoclonus-dystonia (DYT-SGCE, previously referred to as DYT11) with onset in childhood or adolescence is characterized by a combination of myoclonic jerks and dystonic symptoms [1,2,3,4]. These myoclonic jerks are usually alcohol-responsive and often present in the upper body in addition with no or mild to moderate dystonia [5]. Dystonic symptoms of DYT-SGCE mostly present as focal or segmental dystonia manifesting in spasmodic torticollis or writer’s cramp [1,2,6]. Psychiatric comorbidities frequently accompany motor manifestations in DYT-SGCE including alcohol abuse, anxiety-related and obsessive-compulsive disorders [7,8,9,10].

This movement disorder can only be treated symptomatically with systemic pharmacological therapy, e.g., anticholinergic or GABAergic drugs, intramuscular botulinum toxin injection, or deep brain stimulation [11,12,13]. Thus, a better understanding of the pathophysiology is crucial in order to develop disease-modifying treatment options.

DYT-SGCE myoclonus-dystonia is caused by mutations in the *ε-sarcoglycan* gene (*SGCE)* [14]. It is located on chromosome 7q21 and more than 41 different mutations in *SGCE* are known including exon rearrangements, base pair substitutions, deletions or insertions [15,16]. DYT-SGCE is inherited in an autosomal dominant manner with a variable expression and reduced penetrance due to maternal genomic imprinting of the *SGCE* gene [14,17,18,19].

The protein ε-sarcoglycan is a member of the sarcoglycan family of transmembrane glycoproteins with six different isoforms (α-, β-, γ-, δ-, ε-, and ζ-sarcoglycan). These transmembrane proteins are part of the dystrophin-glycoprotein-complex, which is important for the linkage of the cytoskeleton with the extracellular matrix [20]. A brain specific isoform of *SGCE* is broadly expressed in different brain regions, predominantly in the cerebral cortex, the cerebellum and the hippocampus [14,21,22], whereas in the striatum a moderate expression of *SGCE* was found [21].

It was hypothesized that DYT-SGCE dystonia is rather not caused by changes at the neurotransmitter level, but results from alterations in neuronal structures [14,21]. A recent review speculated that loss of ε-sarcoglycan induces neuronal membrane damage leading to calcium accumulation [23]. Calcium homeostasis plays an important role in the regulation of the dopamine D2-receptor [24]. In a ^123^I-IBZM-SPECT (single photon emission computed tomography (SPECT) with the ligand [^123^I]iodobenzamide (IBZM)) study of *SGCE* mutation carriers, a decreased dopamine D2-receptor availability in the striatum could be detected [25]. Reduced striatal D2-receptors were also found in *SGCE* knock-out mice showing myoclonus, impaired motor skills, hyperactivity, anxiety and depression [26]. In addition, these mice exhibited an altered monoamine metabolism with significantly increased striatal dopamine levels and its metabolites as well as inversely decreased serotonin levels [27]. GABAergic networks may also play a crucial pathophysiological role in DYT-SGCE because patients reported improvement of motor symptoms following alcohol administration [7,28,29]. Moreover, studies have demonstrated that the dystrophin-glycoprotein-complex co-localizes with GABAergic synapses and that disturbances of the dystrophin-glycoprotein-complex result in a reduced postsynaptic clustering of GABA_A_ receptors [30,31,32]. 

In a genetic mouse model of *SGCE* deficiency, impaired striatal plasticity was found despite the absence of an obvious motor phenotype [33]. Abnormal nuclear envelopes could be detected in striatal medium spiny neurons in *SGCE* knock-out mice. However, data on striatum-specific *SGCE* conditional knock-out mice suggested that a loss of ε-sarcoglycan in the striatum only contributed to motor deficits while a loss of ε-sarcoglycan in other brain regions might lead to myoclonus and abnormal nuclear envelopes [34]. In accordance with this assumption, it could be shown that the knockdown of *SGCE* in the cerebellum produced motor symptoms with cerebellar involvement in a mouse model of DYT-SGCE [35]. A neuroimaging study in DYT-SGCE patients detected white matter changes in the brain stem suggesting an abnormal function of networks including cerebellum, brain stem and basal ganglia [36]. Taken together, dysfunction of the cerebello-thalamo-cortical and/or striato-pallido-thalamo-cortical pathways was suggested supporting the classification of DYT-SGCE as a neurodevelopmental circuit disorder [23].

In this study, we investigated the molecular and functional phenotype of striatal medium spiny neurons (MSNs) differentiated from DYT-SGCE patient-derived induced pluripotent stem cells (iPSCs) and from healthy controls by adapting two of our previously established protocols [37,38]. To evaluate the functional phenotype of disease-specific and control MSNs, we used calcium imaging and whole-cell patch-clamp recordings. Furthermore, gene expression analysis of voltage-gated Ca^2+^ channel subunits and ionotropic receptor subunits as well as morphometric analysis of MSNs including GABAergic synaptic density were performed to identify novel treatment targets for DYT-SGCE dystonia.

## 2. Results

### 2.1. Differentiation of iPSCs into Medium Spiny Neurons (MSNs)

**Figure 1:**
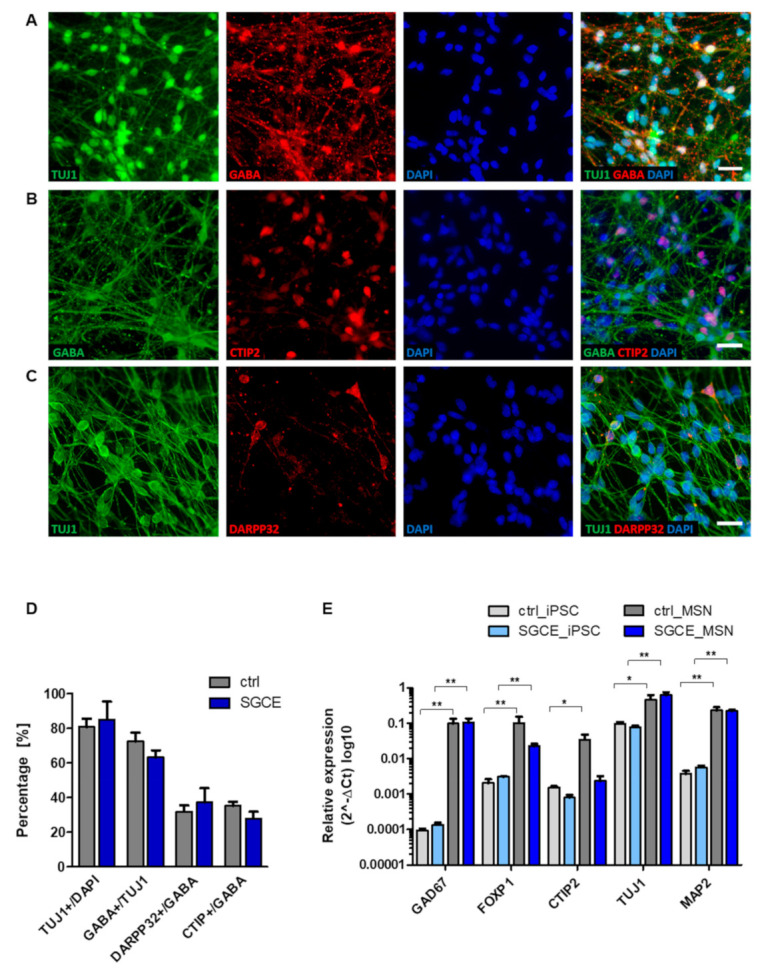
Differentiation of iPSCs derived from DYT-SGCE patients and healthy controls into striatal medium spiny neurons (MSNs). (**A**–**C**) Mature MSNs at day 70 (+/− 7 days) of differentiation expressed the neuronal markers TUJ1, GABA and MSN-specific striatal markers DARPP32 and CTIP2 as shown by representative images of immunofluorescence stainings. Nuclei were stained with 4,6-diamidino-2-phenylindole (DAPI). Scale bar indicates 20 µm. (**D**) About 80% of DAPI-stained MSNs expressed the neuronal marker TUJ1, of which the vast majority were GABA-positive cells. About 30% of GABA-positive cells co-expressed DARPP32 and CTIP2 showing terminal differentiation into striatal MSNs. Quantitative estimation of neuronal and striatal markers was similar for SGCE and control MSNs. Data are presented as means ± SEM from at least two independent differentiations of each cell line. (**E**) Expression analysis of iPSCs and MSNs by quantitative real-time PCR. Compared to iPSCs, SGCE and control MSNs expressed elevated levels of markers for neuronal (FOXP1, TUJ1, MAP2), GABAergic (GAD67) and MSN-specific striatal origin (CTIP2). Expression of neuronal, GABAergic and MSN-specific markers was similar for SGCE and control MSNs. Data are presented as means ± SEM in logarithmic scale (log_10_) from three independent differentiations for SGCE and control lines (* *p* < 0.05, ** *p* < 0.01, parametric t-test or nonparametric Mann–Whitney-U test).

For modeling myoclonus-dystonia in vitro*,* we differentiated MSNs for 70 days from iPSC origin (Figure 1). After plating embryoid bodies (EBs), neural progenitor cells expressed nestin around day 18 during maturation (data not shown). The quantitative immunocytochemical analysis of mature MSNs revealed 81–85% β-tubulin III (TUJ1)-positive neurons of which 63–73% were positive for the neurotransmitter γ-aminobutyric acid (GABA). Approximately ~30% of GABAergic MSNs co-expressed the striatal markers dopamine- and cAMP-regulated neuronal phosphoprotein 32kDa (DARPP32) and COUP TF1-interacting protein 2 (CTIP2) (Figure 1A–D). There was no difference between healthy controls and SGCE MSNs when we quantitatively analyzed the immunofluorescence data. The mRNA expression of neuronal and striatal markers in MSNs showed a significant upregulation compared to the iPSC origin, but was not significantly different for control and SGCE MSNs (Figure 1E). The upregulation of glutamic acid decarboxylase (GAD67), transcription factor forkhead box protein P1 (FOXP1), which is associated with MSN maturation, and the mature neuronal marker microtubule-associated protein 2 (MAP2) confirmed the neuronal and mainly GABAergic phenotype of MSNs. 

### 2.2. Reduced Synaptic Density in SGCE MSNs

**Figure 2:**
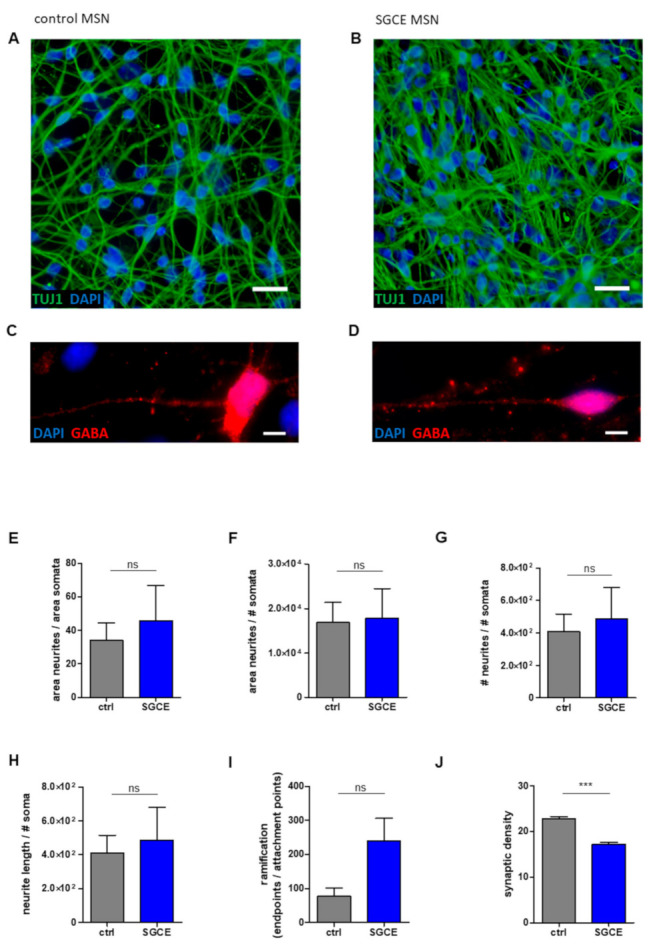
Neuronal morphology and GABAergic synaptic density of MSNs from DYT-SGCE patients and healthy controls. Control (**A**,**C**) and SGCE MSNs (**B**,**D**) were stained for neuronal markers TUJ1 and GABA to analyze neuronal morphology using ImageJ plugin Neurphology and quantify GABAergic synapses by ImageJ plugins SynapCountJ and NeuronJ. Nuclei were stained with DAPI. Scale bar indicates 20 µm (**A**,**B**) and 5 µm (**C**,**D**). Total neurite outgrowth parameters were quantified by normalization of neurites to somata. (**E**) The area of neurites was divided by the area of somata or (**F**) the number of somata (# somata). (**G**) The number of neurites (# neurites) was divided by the number of somata (# somata). (**H**) Total neurite length was obtained by neurite length divided by # somata. All parameters were comparable between SGCE and control MSNs. (**I**) For the expression of total ramification, the number of endpoints of neurites was normalized to the number of attachment points at the soma. The SGCE MSNs showed a tendency of increased ramification compared to control MSNs. (**J**) SGCE MSNs showed a significantly lower amount of GABAergic synapses compared to controls. Abbreviation: ns, not significant. Data are presented as means ± SEM from two to three independent differentiations for SGCE and control lines (*** *p* < 0.001, nonparametric Mann–Whitney-U test).

The morphometric analyses of MSN neurites (using β-tubulin III (TUJ1)-staining, Figure 2A,B) displayed no differences between control and SGCE cell lines after analysis of parameters including neurite length, neurite area and number of neuronal somata per acquired image (Figure 2E–H). Furthermore, no difference of ramification between control and SGCE MSNs was detected by analyzing the number of attachment points of neurites at the soma and endpoints of neurites (Figure 2I). Interestingly, the quantification of GABA-positive boutons in MSNs for the evaluation of synapse formation revealed a significantly reduced (*p* < 0.001) synaptic density in SGCE compared to control MSNs (Figure 2C,D,J). 

### 2.3. Ca^2+^ Signaling in MSNs

#### 2.3.1. Elevated Basal Intracellular Ca^2+^ Levels and Lower Frequency of Spontaneous Ca^2+^ Signals in SGCE MSNs

**Figure 3:**
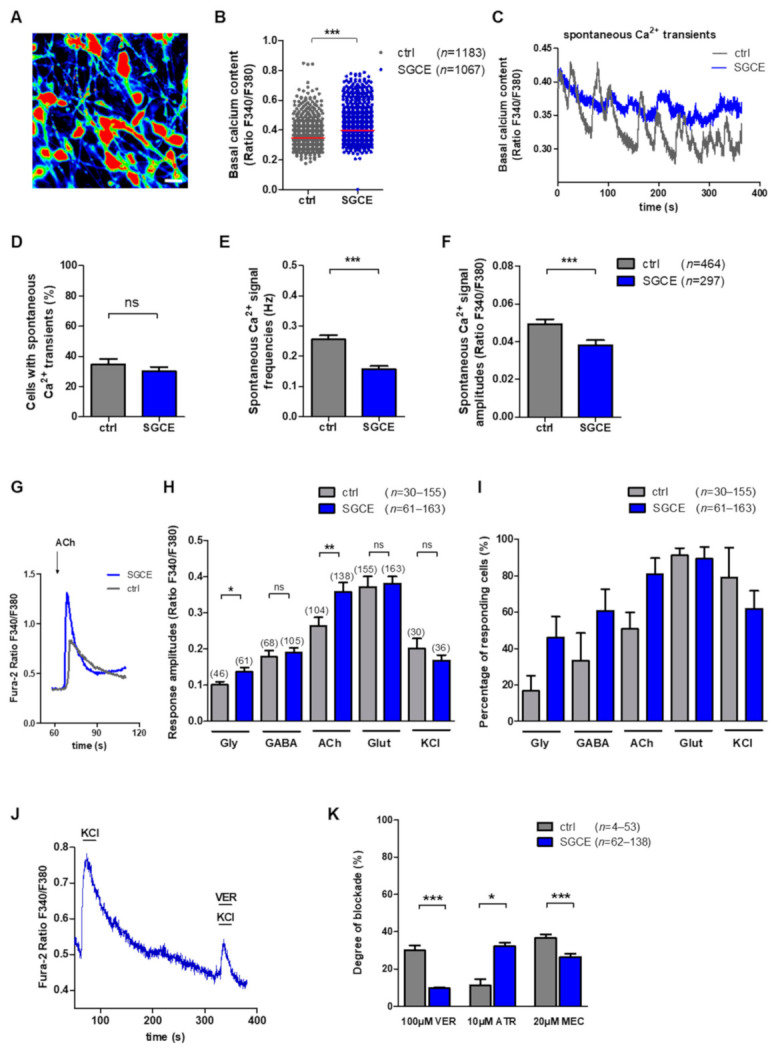
Spontaneous intracellular calcium (Ca^2+^) dynamics, neurotransmitter-induced Ca^2+^ signaling and inhibition of voltage-gated Ca^2+^ channels and acetylcholine receptors in MSNs derived from DYT-SGCE patients and healthy controls. Intracellular Ca^2+^ transients are presented as ratios of the fluorescence signals obtained at 340 and 380 nm (F_340_/F_380_). (**A**) Representative image of Ca^2+^ recordings in SGCE MSNs loaded with Fura-2. Scale bar indicates 30 µm. (**B**) Basal intracellular Ca^2+^ levels were significantly elevated in SGCE (*n* = 1067) compared to control MSNs (*n* = 1183, *** *p* < 0.001, nonparametric Mann–Whitney-U test). (**C**) Representative traces of spontaneous Ca^2+^ peaks of control and SGCE MSNs. (**D**) The percentage of cells showing spontaneous Ca^2+^ transients was not significantly different between both MSN groups (~38%, unpaired *t*-test). (**E**) Spontaneous Ca^2+^ signal frequencies (*** *p* < 0.001, nonparametric Mann–Whitney-U test) and (**F**) amplitudes were significantly lower in SGCE (*n* = 297) compared to control MSNs (*n* = 464, *** *p* < 0.001, nonparametric Mann–Whitney-U test). Data are presented as means ± SEM from at least three independent differentiations for SGCE and control lines. (**G**) Representative traces of intracellular Ca^2+^ changes of Fura-2 loaded control and SGCE neurons induced by separate application of acetylcholine (ACh, 100 µM). (**H**) Cytosolic Ca^2+^ response amplitudes upon separate applications of the neurotransmitters glycine (Gly,100 µM), γ-aminobutyric acid (GABA, 100 µM), acetylcholine (ACh, 100 µM) and glutamate (Glut, 50 µM) normalized to the basal Ca^2+^ level of MSNs (*n* = number of cells) showed significantly higher Ca^2+^ peaks during application of glycine and acetylcholine in SGCE compared to control MSNs (* *p* < 0.05, ** *p* < 0.001, nonparametric Mann–Whitney-U test). (**I**) Percentage of cells responding to neurotransmitter applications with Ca^2+^ rises shown in subfigure H was similar in SGCE and control MSNs (nonparametric Mann–Whitney-U test). (**J**) Representative trace of intracellular Ca^2+^ changes in a Fura-2 loaded SGCE MSN induced by separate application of 50 mM KCl followed by KCl and verapamil (100 µM, VER). (**K**) Significantly lower blockade of KCl-induced Ca^2+^ transients by separate application of verapamil (100 µM, blocks voltage-gated Ca^2+^ channels) in SGCE MSNs (control *n* = 53, 29.9% blockade; SGCE *n* = 138, 9.7% blockade). Application of either atropine (10 µM, ATR, muscarinic acetylcholine receptor antagonist, control *n* = 4, 11.2% blockade; SGCE *n* = 62, 32.0% blockade) or mecamylamine (20 µM, MEC, nicotinic acetylcholine receptor antagonist, control *n* = 48, 36.6% blockade; SGCE *n* = 78, 26.4% blockade) suppressing Ca^2+^ peaks evoked by 100 µM acetylcholine application in MSNs. Data are presented as means ± SEM from at least three independent differentiations for SGCE and control lines for neurotransmitter applications. Abbreviation: ns, not significant. Data were recorded from two independent differentiations for blocking of voltage-gated Ca^2+^ channels and acetylcholine receptors during Ca^2+^ signaling (* *p* < 0.05, ** *p* < 0.01, *** *p* < 0.001, nonparametric Mann–Whitney-U test).

A representative image of intracellular Ca^2+^ recordings of Fura-2 loaded SGCE MSNs is shown in Figure 3A. The SGCE MSNs showed significantly elevated basal intracellular Ca^2+^ levels compared to controls (SGCE 0.39 ± 0.001, control 0.35 ± 0.001, *p* < 0.001, Figure 3B). A representative image of spontaneous Ca^2+^ transients of MSNs is shown in Figure 3C. The percentage of MSNs with spontaneous Ca^2+^ signals was not significantly different between SGCE (30.2 ± 2.8%) and control MSNs (34.8 ± 3.6%, Figure 3D). However, the frequency (SGCE 0.157 ± 0.011 Hz, control 0.255 ± 0.014 Hz, *p* < 0.001, Figure 3E) and the amplitudes (SGCE 0.038 ± 0.003, control 0.049 ± 0.003, *p* < 0.001, Figure 3F) of spontaneous Ca^2+^ transients were significantly lower in SGCE compared to control MSNs.

#### 2.3.2. Elevated Ca^2+^ Amplitudes upon Glycine and Acetylcholine Application in SGCE MSNs

Representative traces of intracellular Ca^2+^ transients in Fura-2 loaded control and SGCE MSNs induced by separate application of acetylcholine (100 µM) are shown in Figure 3G. Analysis of neurotransmitter-induced Ca^2+^ signaling revealed that SGCE MSNs showed significantly elevated amplitudes of Ca^2+^ transients in response to application of glycine (SGCE 0.14 ± 0.013, control 0.10 ± 0.009, *p* < 0.05) and acetylcholine (SGCE 0.19 ± 0.013, control 0.27 ± 0.023, *p* < 0.01), whereas Ca^2+^ peaks elicited by GABA and glutamate application were similar in both groups (Figure 3H). The percentage of cells responding with Ca^2+^ rises was similar upon individual neurotransmitter applications in both MSN groups (Figure 3I).

#### 2.3.3. Blocking of Ca^2+^ Amplitudes after Application of Antagonists for Voltage-Gated Ca^2+^ Channels and Acetylcholine Receptors in MSNs

The viability and excitability of MSNs was evaluated by application of depolarizing KCl and was similar for control and SGCE MSNs. A representative trace of blocking Ca^2+^ transients in Fura-2 loaded SGCE MSN by verapamil (100 µM) following applications of KCl (50 mM) is shown in Figure 3J. The drug verapamil inhibits voltage-gated Ca^2+^ channels nonspecifically and the blocking degree in percentage was significantly lower in SGCE compared to control MSNs (SGCE 9.7 ± 0.6%, *n* = 138, control 29.9 ± 2.8%, *n* = 52, *p* < 0.001, Figure 3K). The blocking effect of atropine was significantly higher in SGCE compared to control MSNs (SGCE 32.0% ± 2.1%, *n* = 62, control 11.2 ± 3.3%, *n* = 4, *p* < 0.05, Figure 3K). In contrast, the blocking degree of mecamylamine was markedly lower in SGCE MSNs (SGCE 26.4% ± 1.8%, *n* = 78, control 36.6 ± 2.0%, *p* < 0.001, Figure 3K). 

### 2.4. Expression of Voltage-Gated Ca^2+^ Channels and Ionotropic Receptor Subunits in MSNs

**Figure 4:**
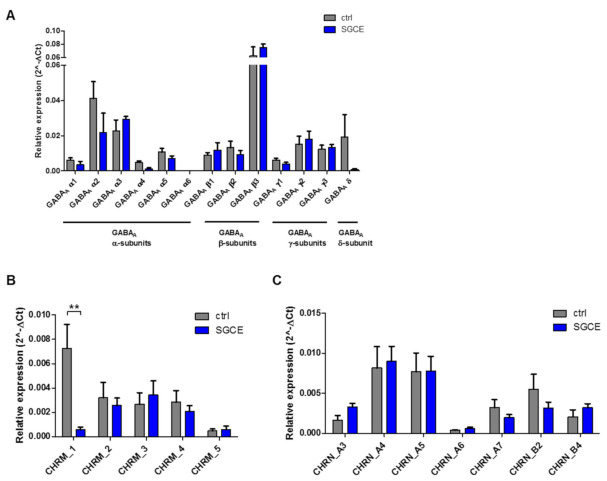
Expression analysis of ionotropic receptor subunits in MSNs from DYT-SGCE patients and healthy controls by quantitative real-time PCR. (**A**) Expression of genes encoding γ-aminobutyric acid type A (GABA_A_) receptor subunits α (1–6), β (1–3), γ (1–3) and ∂ was similar in SGCE compared to control MSNs. (**B**) Expression of the muscarinic acetylcholine receptor subunit M_1_ (encoded by *CHRM1*) was significantly lower in SGCE MSNs, whereas the expression of the subunits M_2-_M_5_ (encoded by *CHRM1-CHRM5*) was similar in SGCE and control MSNs. (**C**) Expression of nicotinic acetylcholine receptor subunits α (3–7), β2 and β4 (encoded by *CHRNA3-CHRNA7, CHRNB2, CHRNB4*) was not significantly different in SGCE and control MSNs. Data are presented as means ± SEM from three independent differentiations for SGCE and control lines (** *p* < 0.01, nonparametric Mann–Whitney-U test).

Genomic expression levels of genes encoding voltage-gated Ca^2+^ channels Ca_v_1.2–1.3, Ca_v_2.1–2.3 and Ca_v_3.1–3.3 were comparable in both MSN groups (data not shown). The expression of GABA_A_ receptor subunits α, β, γ and δ was not significantly different in SGCE and control MSNs. All MSNs showed predominant expression of synaptic GABA_A_ receptor subunits, particularly β3 followed by α2 and α3, whereas subunits typical for extra-synaptic receptors (α4, α6 and δ) were barely expressed in SGCE MSNs (Figure 4A). The expression of muscarinic acetylcholine receptor (mAChR) subunit M_1_ was significantly lower for SGCE MSNs compared to control MSNs. The genomic expression of mAChR subunits M_2_-M_5_ (encoded by genes *CHRM2-CHRM5*) was similar in SGCE and control MSNs with lowest expression of *CHRM5* (Figure 4B). Levels of transcripts encoding for nicotinic acetylcholine receptor (nAChR) subunits α (3–7), β2, β4 (encoded by genes *CHRNA3-CHRNA7, CHRNB2* and *CHRNB4*) were equal in SGCE and control MSNs (Figure 4C). Predominantly, *CHRNA4* and *CHRNA5* were expressed in MSNs.

### 2.5. Properties of Voltage-Gated Ion Channels, Synaptic Activity and Action Potentials in MSNs

**Figure 5:**
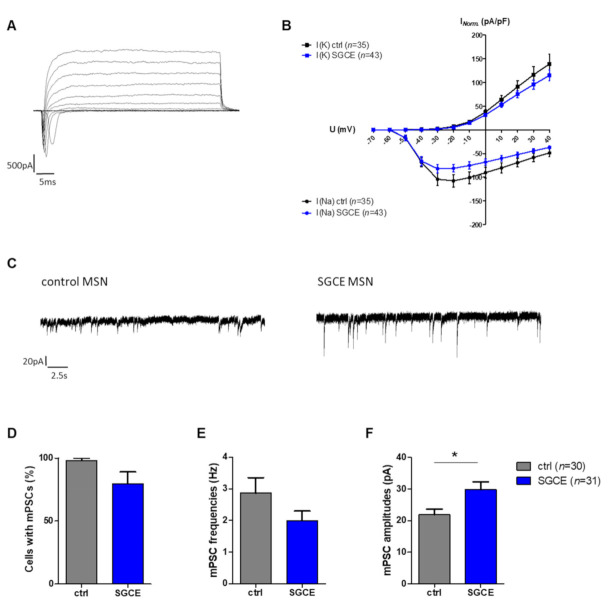
Voltage-gated ion currents and action potential (AP) recordings of MSNs derived from DYT-SGCE patients and healthy controls. (**A**) Voltage-gated sodium inward and potassium outward currents of SGCE MSN at day 70 in vitro were recorded in whole-cell voltage-clamp mode by depolarizing steps in increments of 10 mV from a holding potential of −70 to 40 mV. (**B**) Ion currents normalized for individual cell sizes based on capacitances of the cell membrane (pA/pF) were not significantly different between SGCE (*n* = 43) and control MSNs (*n* = 35, two-way ANOVA with Bonferroni post-hoc test). (**C**) Traces of miniature postsynaptic currents (mPSC), indicating spontaneous synaptic events from MSN afferents, were recorded in whole-cell voltage-clamp mode at a holding potential of −70 mV. (**D**) Percentage of cells with mPSCs and (**E**) mPSC frequencies were not significantly different for SGCE (*n* = 31) and control MSNs (*n* = 30, nonparametric Mann–Whitney-U test). (**F**) The mPSC amplitudes were significantly higher in SGCE compared to control MSNs (* *p* < 0.05, nonparametric Mann–Whitney-U test). Data are presented as means ± SEM from at least two independent differentiations for SGCE and control lines.

Whole-cell patch-clamp recordings from MSNs of DYT-SGCE patients and healthy controls were performed to assess voltage-gated ion channels, action potential properties and synaptic activity (Table 1). SGCE and control MSNs showed sodium inward and potassium outward currents upon depolarizing steps in increments of 10 mV from a holding potential of −70 to 40 mV (Figure 5A). After normalization of ion current amplitudes for individual cell sizes based on capacitances of the cell membrane (pA/pF), both groups did not significantly differ (Figure 5B). 

#### 2.5.1. Larger mPSC Amplitudes in SGCE MSNs

Measurements of spontaneous synaptic activity of MSNs revealed that the percentage of cells with miniature postsynaptic currents (mPSCs) was lower in SGCE (79.7%) compared to control MSNs (98.2%), however, without significant difference (Figure 5C,D). The mPSC frequencies were similar in both groups, while mPSC amplitudes were significantly higher in SGCE than in control MSNs (SGCE 29.8 ± 2.4 pA, control 21.9 ± 1.8 pA, *p* = 0.011, Figure 5E,F).

#### 2.5.2. Elevated Amplitudes of Evoked Action Potentials in SGCE MSNs

**Figure 6:**
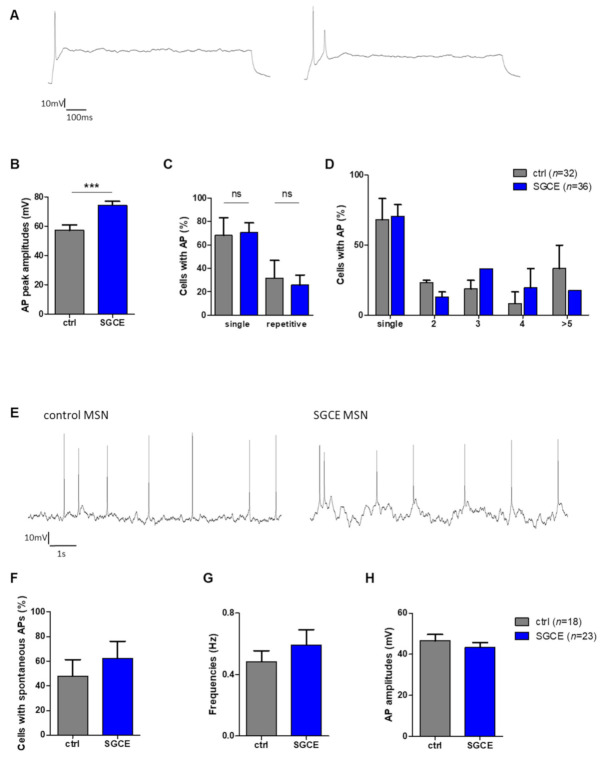
Evoked and spontaneous action potentials (APs) of MSNs from DYT-SGCE patients and healthy controls. (**A**) Example of SGCE MSNs firing single and repetitive APs upon depolarization in current-clamp mode from a holding potential of approximately −70 mV. (**B**) Evoked AP amplitudes were significantly elevated in SGCE (*n* = 36) compared to control MSNs (*n* = 32, *** *p* < 0.001, unpaired *t*-test). (**C**) Percentages of cells with single or repetitive APs were not significantly different between MSNs of both groups (nonparametric Mann–Whitney-U test). (**D**) Percentages of neurons with single or multiple (2, 3, 4, ≥ 5) APs were not significantly different between MSNs of both groups (two-way ANOVA with Bonferroni post hoc test). (**E**) Spontaneous AP firing was measured at a holding potential of approximately −60 mV. (**F**) Percentage of cells with spontaneous APs, (**G**) frequencies and (**H**) amplitudes of spontaneous APs were comparable for SGCE (*n* = 23) and control MSNs (*n* = 18, nonparametric Mann–Whitney-U test). Abbreviation: ns, not significant. Data are presented as means ± SEM from at least two independent differentiations for SGCE and control lines.

Single action potentials (APs) elicited by depolarizing current injections from a holding potential of approximately −70 mV were fired from the majority of control (68%) and SGCE MSNs (71%, Table 1, Figure 6A). The percentage of MSNs with repetitive APs after evoked spiking was also similar for SGCE (29%) and control group (32%, Table 1, Figure 6A,C,D). The amplitudes of evoked APs were significantly (*p* < 0.001, Figure 6B) elevated for SGCE (74.3 ± 2.6 mV) compared to control MSNs (57.2 ± 3.7 mV). In addition, AP duration was significantly shorter in SGCE compared to control MSNs (SGCE 2.3 ± 0.1 ms, control 3.2 ± 0.4 ms, *p* < 0.05, Table 1). The amplitude of after-hyperpolarization (AHP) was significantly elevated in SGCE MSNs (SGCE 8.2 ± 0.9 mV, control 6.0 ± 1.3 mV, *p* < 0.05, Table 1), whereas the time to peak AHP was comparable (SGCE 26.0 ± 3.1 ms, control 20.9 ± 1.3 ms, Table 1). The number of SGCE cells with spontaneous APs was higher, but not significantly different compared to control MSNs (SGCE 62.3 ± 13.9%, control 48.0 ± 13.3%, Figure 6E,F). Furthermore, the frequency (SGCE 0.60 ± 0.1 Hz, control 0.49 ± 0.07 Hz) and amplitude of spontaneous APs was similar for both groups (SGCE 43.3 ± 2.3 mV, control 46.5 ± 3.2 mV, Figure 6G,H).

## 3. Discussion

To better understand the pathophysiology of DYT-SGCE, we investigated iPSC-derived striatal MSNs of DYT-SGCE patients and healthy controls after 70 days of differentiation adapting previously established in vitro iPSC differentiation protocols towards striatal neurons [37,38]. Our preliminary data showed in patient neurons the selective expression of the mutated paternal SGCE allele due to maternal imprinting and dramatically reduced SGCE protein levels confirming the suitability of this iPSC model for myoclonus-dystonia research [19].

In calcium imaging recordings, the basal intracellular calcium levels were significantly elevated in SGCE-mutant compared to control MSNs. Besides, the frequency and amplitude of spontaneous calcium signals were lower in SGCE MSNs. A previous investigation demonstrated that accumulated intracellular Ca^2+^ accompanied the absence of dystrophin in neurons [39]. It can be assumed that the same processes might occur when a protein of the dystrophin-glycoprotein-complex other than dystrophin, such as ε-sarcoglycan, is affected [23]. Therefore, calcium homeostasis is likely to play an important role in the course of this genetic form of myoclonus-dystonia. Ca^2+^ signals are less suppressed in SGCE than in control MSNs after unspecific blockade of voltage-gated Ca^2+^ channels by application of verapamil, although the expression of these channels is comparable. Two case reports described that myoclonic dystonia [40] or multifocal myoclonus [41] could be induced by verapamil.

Recordings of calcium signaling after neurotransmitter application revealed elevated calcium amplitudes upon glycine and acetylcholine applications in SGCE MSNs. Ca^2+^ signals evoked by acetylcholine were significantly less blocked by the nicotinic acetylcholine receptor antagonist mecamylamine in SGCE compared to control MSNs. In contrast, the muscarinic acetylcholine receptor antagonist atropine inhibited significantly stronger after acetylcholine application in SGCE MSNs. These results may suggest an increased signaling via the muscarinic acetylcholine receptor in DYT-SGCE MSNs compared to healthy controls, which could be accompanied by a reduced transmission via the nicotinic acetylcholine receptor. In line with this, a medication with anticholinergic drugs such as trihexyphenidyl that is a muscarinic acetylcholine receptor blocker, could improve motor symptoms in DYT-SGCE [42,43,44,45]. So it could be hypothesized that cholinergic signaling, especially via muscarinic acetylcholine receptors, plays an important role in DYT-SGCE. In striatal MSNs of DYT-TOR1A, anticholinergic drugs inhibiting the M1 muscarinic acetylcholine receptor, such as trihexyphenidyl, have been shown to improve symptoms [46]. Besides, it was demonstrated that extrapyramidal syndromes induced by antipsychotic drugs could be treated successfully by selective M1 antagonists [47].

However, gene expression analysis of transcripts encoding for neuronal muscarinic acetylcholine receptor (mAChR) subunits revealed a significantly lower expression of *CHRM1* (encoding for mAChR subunit M_1_) in patient-derived SGCE MSNs compared to the control group. In contrast, a previous study using a dystonic hamster model linked increased expression of the acetylcholine receptor subunit M_1_ in the striatum to the involvement of the cholinergic system resulting in abnormal striatal plasticity [48]. As it would have been expected from previous studies, *CHRM5* was expressed very low in human MSNs whereas transcripts encoding for other mAChR subtypes M_2_-M_4_ were more abundant [44,49]. The discrepancy between elevated acetylcholine-induced Ca^2+^ signals and decreased expression of *CHRM1* in SGCE MSNs may in part be explained by posttranslational changes of acetylcholine receptors resulting in a compensatory higher Ca^2+^ influx. Regarding the expression of nicotinic acetylcholine receptor subunits (nAChRs), comparable ratios as known from the literature were also detected in our iPSC-derived MSNs, with lower expression levels of *CHRNA6* and higher levels of *CHRNA5* (encoding for nAChR subunits α6 and α5) in relation to iPSC-derived neurons comprising primarily of GABAergic and glutamatergic cells [50]. The most widely expressed neuronal nAChR subtype in the brain is heteromeric α4β2 [44,51]. Possibly, altered afferents of striatal cholinergic interneurons or posttranslational modifications of acetylcholine receptors in SGCE MSNs may account for some of the differences in cholinergic signaling. In line with this, changes in acetylcholine receptors and release were seen in the hippocampus of dystrophin-deficient mice [52,53]. A favorable pathogenic hypothesis is that DYT-SGCE may be caused by altered neuronal structures, especially in the dystrophin-glycoprotein-complex [22,23,39].

Interestingly, SGCE MSNs displayed significantly reduced GABAergic synaptic density in comparison to control MSNs. However, the expression of the genes encoding GABA_A_ receptor subunits and GABAergic calcium signaling were comparable in SGCE and control MSNs. The reduced neuronal GABAergic synaptic density in striatal MSNs seen in our study could be one pathophysiological cause for the improvement of symptoms after ingestion of alcohol [28,29,45] or likewise benzodiazepines [1,42,45], which both enhance GABAergic transmission. Even earlier, it was suggested that the striatum plays an important pathophysiological role in DYT-SGCE but other brain regions seem to be also involved in the pathogenesis [27,33,34]. In accordance with the assumption that the striatum as part of the basal ganglia could be important for the pathogenesis of DYT- SGCE, deep-brain stimulation of the internal globus pallidus and the ventral intermediate thalamic nucleus improved symptoms of DYT-SGCE patients [54,55,56]. A dysfunction of the cerebello-thalamico-cortical and striato-pallido-thalamo-cortical pathway, potentially due to a GABAergic deficit, is assumed to be one of the main causes of DYT-SGCE [5,23,45].

In conclusion, the differentiation of iPSCs towards striatal MSNs provides a feasible in vitro model of DYT-SGCE myoclonus-dystonia and gives insights into the functional phenotype. Despite our small number of cell lines, our data might contribute to an improved understanding of the pathophysiology of DYT-SGCE and may help to advance the development of new therapeutic strategies. However, future studies that analyze data from multiple DYT11 mutations including controls with an isogenic background are needed to determine what is the common cellular pathology.

## 4. Materials and Methods

### 4.1. Cultivation of Human iPSC Lines

Both patient-derived DYT-SGCE iPSC lines were reprogrammed using retroviruses and published recently [19] (Table 2). Genotype and phenotype of DYT-SGCE myoclonus-dystonia patients were examined recently [9] indicating patient SGCE_1 (L-5007) with point mutation in *SGCE* c.298T>G, p.W100G and patient SGCE_2 (L-6074) with c.304C>T in *SGCE*, p.R102X. Both patients had brachial dystonia and myoclonus with 6 years of age at the onset of motor symptoms. No signs of psychiatric symptoms were reported [9].

The two age- and sex-matched healthy controls were also analyzed previously [57,58] (Table 2). For the sake of visual clarity, we decided to illustrate figure bar graphs with pooled data of control and SGCE cell lines, respectively. Data of these healthy control lines have also been included in another manuscript [59].

During expansion, the iPSCs were cultivated feeder-free in mTeSR medium (Stemcell Technologies, Vancouver, BC, Canada) as colonies and detached using 0.5 mM EDTA for splitting every 4–7 days [60]. 

### 4.2. Differentiation of iPSC Lines into Striatal Medium Spiny Neurons (MSNs)

The striatal differentiation of iPSCs into medium spiny neurons (MSNs) was adapted from previous protocols published by Stanslowsky et al. [37] and Capetian et al. [38]. Briefly, feeder-free iPSC colonies were detached and suspended in mTeSR (Stemcell Technologies, Vancouver, BC, Canada) supplemented with 1 µM dorsomorphin (Tocris, Bio-Techne, Minneapolis, MN, USA), 10 µM SB-431542 (Tocris, Bio-Techne, Minneapolis, MN, USA), 1 µM IWP2 (Merck, Darmstadt, Germany), and 10 µM Rho kinase (ROCK) inhibitor Y27632 (Stemcell Technologies, Vancouver, BC, Canada) to form free-floating embryoid bodies (EBs) at day 0. On day 2, medium was replaced with 1:1 mTeSR/N2 medium (Knockout-DMEM/F-12, with 1:100 N2 supplement, Thermo Fisher Scientific, Waltham, MA, USA and 1% penicillin, streptomycin, L-glutamine, Thermo Fisher Scientific, Waltham, MA, USA) supplemented with the above-mentioned concentrations of dorsomorphin, SB-431542, and IWP2. On day 4, N2 medium was supplemented with dorsomorphin, SB-431542, IWP2, and 0.2 µM purmorphamine (PMA; Enzo Life Sciences, Lörrach, Germany). On day 6 and 8, medium was replaced with N2 medium supplemented with 1 µM IWP2 and 0.2 µM PMA. On day 10, only N2 medium without supplements was used. On day 12, EBs were plated and kept in N2B27 maturation medium (1:1 DMEM/F-12 and Neurobasal medium containing 1:200 N2, 1:100 B27 without vitamin A, Thermo Fisher Scientific, Waltham, MA, USA) and 1% penicillin, streptomycin, L-glutamine) with 20 ng/mL brain-derived neurotrophic factor (BDNF; PeproTech, Cranbury, NJ, USA), 10 ng/mL glial cell line-derived neurotrophic factor (GDNF; PeproTech, Cranbury, NJ, USA) and 50 µM dibutyryl-cAMP (dbcAMP; Sigma-Aldrich, St. Louis, MO, USA). Once the outgrowing cells reached full confluency, they were replated on laminin/poly-DL-ornithine hydrobromide (Thermo Fisher Scientific, Waltham, MA, USA) coated dishes for terminal differentiation. Maturation medium was changed every other day. Fully differentiated neuronal cells were characterized after 70 days ± 7 days. A total of two to three independent MSN differentiations of each iPSC line (two control and two SGCE lines) were analyzed. 

### 4.3. Immunocytochemistry

Immunofluorescence stainings were performed on day 16 to evaluate outgrowth of neuronal cells from plated EBs and after day 70 ± 7 days to quantify the amount of terminally differentiated MSNs. Cells were fixed for 20 min with 4% paraformaldehyde (PFA, Sigma-Aldrich, St. Louis, MO, USA), washed and incubated for 60 min with blocking solution (5% goat serum, 1% bovine serum albumin (BSA, Sigma-Aldrich, St. Louis, MO, USA), 0.3% Triton X-100 (Sigma-Aldrich, St. Louis, MO, USA) diluted in PBS)). The following primary antibodies were used: rabbit polyclonal anti-GABA (γ-aminobutyric acid, 1:1000, Sigma-Aldrich, St. Louis, MO, USA), mouse anti-GABA (1:500, Abcam, Cambridge, MA, USA), rabbit polyclonal anti-TUJ1 (β-tubulin III, 1:1000, Abcam, Cambridge, MA, USA), mouse IgG2a anti-TUJ1 (1:1000, Abcam, Cambridge, MA, USA), rabbit polyclonal anti-DARPP32 (dopamine and cAMP-regulated neuronal phosphoprotein 32kDa, 1:100, Abcam, Cambridge, MA, USA), rat IgG2a anti-CTIP2 (COUP TF1-interacting protein 2, 1:300, Abcam, Cambridge, MA, USA), mouse IgG1 anti-nestin (1:300, R&D Systems, Minnesota, MN, USA). Primary antibodies were diluted in blocking solution and incubated with the cultures overnight at 4 °C. After washing, cells were incubated with the respective secondary antibodies (AlexaFluor 488 or 555, goat anti-mouse, goat anti-rabbit, goat anti-rat, 1:1000, Thermo Fisher Scientific, Waltham, MA, USA) for 2 h at room temperature. Mounting medium was supplemented with 0.1% DAPI (4,6-diamidino-2-phenylindole, 10 mg/mL, Thermo Fisher Scientific, Waltham, MA, USA). To check for background staining, primary antibodies were omitted for one additional coverslip for each staining combination per differentiation. Specimens were visualized with a fluorescence microscope BX61 (Olympus, Shinjuku, Japan) and DP72 camera (Olympus, Shinjuku, Japan) using the analysis software Cell^F^ (Olympus, Shinjuku, Japan). Four random visual fields per coverslip were taken from two independent differentiations for each control and SGCE line. Cells were counted manually using ImageJ (NIH, Bethesda, MD, USA). Representative images were processed using ImageJ. 

### 4.4. Analysis of Neuronal Morphology and Synaptic Density

Cells were stained with primary antibodies mouse anti-TUJ1 (β-tubulin III, 1:1000, Abcam, Cambridge, MA, USA) and rabbit anti-GABA (1:1000, Sigma-Aldrich, St. Louis, MO, USA). Pictures for image analysis were taken at 40-fold magnification using a fluorescence microscope BX61 (Olympus), DP72 camera (Olympus, Shinjuku, Japan), and software Cell^F^ (Olympus, Shinjuku, Japan). ImageJ-based plugin NeurphologyJ [61] was used to quantify somata, neurites, attachment points, and endpoints as described previously [37,59]. Briefly, the plugin uses two different thresholds for somata and neurites, as well as the information of the approximate neurite thickness for detection and quantification of number of somata and neurites, the area of somata and neurites, and the total neurite length within an image. Those parameters were used to normalize neurites to somata to obtain a quantitative expression for total neurite outgrowth within an image. Similarly, the endpoints of a neurite were normalized to soma attachment points to receive an expression for ramification. For quantification of GABAergic synaptic density, the ImageJ plugins SynapCountJ (version 2.0) and NeuronJ (version 1.4.3) [62] were used as described previously [37,63]. In brief, NeuronJ was used to trace a neurite and this tracing was applied to SynapCountJ, in which GABA-positive spots along the tracing were detected within an intensity threshold. In total, 10–15 images from two independent differentiations were analyzed for each control and SGCE line. 

### 4.5. Calcium Imaging

To measure intracellular calcium transients, MSNs of three to four independent differentiations (70 days ± 7 days) per iPSC line were loaded with the membrane permeable fluorescent dye Fura 2-AM (Sigma-Aldrich, St. Louis, MO, USA). The MSNs were incubated for 20 min at 37 °C with Fura 2-AM in a standard bath solution (containing 140 mM NaCl, 5 mM KCl, 2 mM CaCl_2_, 10 mM glucose, and 10 mM HEPES, adjusted to pH 7.4 with NaOH). To image intracellular calcium, Fura 2-AM loaded MSNs were excited at wavelengths of 340/380 nm and monitored every 300 ms. Recordings were performed on an upright microscope Axioskop 2 FS plus (Carl Zeiss MicroImaging GmbH, Göttingen, Germany) connected to a Till Vision Imaging System (TILL Photonics, Gräfelfing, Germany). Emitted fluorescence was collected by a charge-coupled device (CCD) camera as described previously [59,64]. To monitor spontaneous intracellular Ca^2+^ changes, MSNs with a preferably multipolar dendritic morphology were imaged for 6 min. Further, the neurotransmitters glycine (100 μM), GABA (100 μM), acetylcholine (100 μM), and glutamate (50 μM) were applied to MSNs after 1 min baseline condition and the provoked intracellular Ca^2+^ changes were recorded for 1 min followed by perfusion with bath solution. Note, glycine- and GABA-induced Ca^2+^ peaks suggest a slight depolarizing effect of these neurotransmitters in some cells, most likely due to a high intracellular chloride concentration [65,66]. Recordings were terminated by application of KCl (50 mM) to induce neuronal depolarization and ensure the viability and excitability of the recorded cells. After background subtraction, the 340/380 nm excitation ratio for Fura 2-AM was calculated which increases as a function of the cytosolic free Ca^2+^ concentration ([Ca^2+^]_i_). For analysis of spontaneous Ca^2+^ transients, only Ca^2+^ signals with a 340/380 nm excitation ratio of Fura 2-AM of ≥ 0.02 of individual MSNs were used. For analysis of response amplitudes after neurotransmitter application, only Ca^2+^ signals with a 340/380 nm excitation ratio of Fura 2-AM ≥ 0.05 of individual MSNs were used [59]. Additional experiments in Fura-2 loaded MSNs were performed in which verapamil (100µM, Sigma-Aldrich, St. Louis, MO, USA, to block voltage-gated calcium channels), diluted in a bath solution with KCl (50 mM), was applied to MSNs after 1 min baseline condition. Furthermore, experiments with separate application of either atropine (10 µM, Sigma-Aldrich, St. Louis, MO, USA, muscarinic acetylcholine receptor antagonist) or mecamylamine (20 µM, Sigma-Aldrich, St. Louis, MO, USA, nicotinic acetylcholine receptor antagonist) diluted in a bath solution with acetylcholine (100 µM) to Fura-2 loaded MSNs were recorded. The calcium peaks of each individual experiment were used to calculate the percentage of blocking for each antagonist. Per independent differentiation, at least two coverslips with 10–40 MSNs from two control lines and two SGCE lines were investigated. 

### 4.6. Quantitative Real-Time PCR

For RNA extraction, iPSCs and differentiated MSNs (collected at day 70 days ± 7 days) were processed using RNeasy Mini Kit (Qiagen, Venlo, The Netherlands) including a column DNase digestion. The quality of total RNA was checked by Nanodrop analysis (Nanodrop Technologies, Wilmington, DE, USA). A total of 250 ng of RNA was transcribed into cDNA using QuantiTect reverse transcription kit (Qiagen). Quantitative real-time PCR reaction analysis was performed [61] using Power SYBR PCR Green Master Mix (Thermo Fisher Scientific, Waltham, MA, USA) in a StepOnePlus cycler (Thermo Fisher Scientific, Waltham, MA, USA) under following conditions: 95 °C for 10 min followed by 40 cycles of 95 °C for 15 s and 60 °C for 1 min. Per reaction, a total amount of 1.75 ng RNA transcribed to cDNA was analyzed and 1.75 µM of forward and reverse primers were used. As recommended by the MIQE-guidelines [67], specificity of PCR products was ensured by melting curve analysis and equal PCR efficiency of all primer pairs was validated by serial dilutions of cDNA. For the following primer pairs and PCR products, the PCR efficiency or detection limit Ct < 30 were not meeting the quality criteria by the MIQE guidelines and were not included in further analysis: primer pairs for target genes *CHRNA1, CHRNA2, CHRNB1, CHRNB3* encoding respective nicotinic acetylcholine receptor (nAChR) subunits. The threshold cycle (Ct) values of target genes were normalized to expression of endogenous references *beta2-microglobulin (B2M)*, *glyceraldehyde 3-phosphate dehydrogenase (GAPDH)* and *β-actin* with the following formula: [Ct (target) − Ct (reference) = ΔCt]. Gene expression of three independent differentiations per cell line (two control lines and two SGCE lines), each in triplicate, were used for analysis and illustrated as means ± standard error of the mean (SEM). For a list of all primer sequences of GABA_A_ receptor [66], voltage-gated calcium channel, nicotinic and muscarinic acetylcholine receptor subunits see Supplementary Tables S1–S4. 

### 4.7. Electrophysiology

Electrophysiological patch-clamp measurements of MSNs were performed after 70 days ± 7 days of differentiation in vitro from at least three to four independent differentiations per iPSC line as described previously [37,59,64]. To identify MSNs, only medium-sized multipolar neurons were used for analyses. This morphological selection approach had previously yielded >90% of DARPP-32 positive MSNs in whole-cell recordings [38]. Patch-clamp pipettes with final resistances of 3–4 MΩ were filled with an internal solution (153 mM KCl, 1 mM MgCl_2_, 10 mM HEPES, and 5 mM EGTA, adjusted to pH 7.3 with KOH, 305 mOsm). The external bath solution contained 142 mM NaCl, 8 mM KCl, 1 mM CaCl_2_, 6 mM MgCl_2_, 10 mM glucose, and 10 mM HEPES, adjusted to pH 7.4 with NaOH, 325 mOsm). Whole-cell currents were low-pass filtered at 2.9 kHz, digitized at 10 kHz using an EPC-10 amplifier (HEKA Elektronik, Harvard Bioscience, MA USA), and analyzed with PatchMaster and FitMaster software (HEKA). Sodium and potassium ion currents were elicited by depolarizing voltage steps in increments of 10 mV from a holding potential of −70 to 40 mV. Miniature postsynaptic currents (mPSCs), indicating mostly GABAergic synaptic activity in MSNs [37,38], were acquired at a holding potential of −70 mV in voltage-clamp mode. For quantitative analysis only mPSCs amplitudes between 10–100 pA were measured to exclude noise artefacts (<10 pA) and action potential activity (>100 pA) of the recorded neuron. Spontaneous and evoked action potentials (APs) were recorded in current-clamp mode at holding potentials of −50 to −70 mV. For recordings, MSNs from at least two independent differentiations from two control lines and two SGCE lines were investigated.

### 4.8. Statistical Analyses

Statistical analysis was performed using GraphPad Prism 5 Software (GraphPad Software, San Diego, CA, USA). All data of MSNs from healthy controls and SGCE lines were pooled in control and SGCE groups for the respective experiments and are presented as mean ± standard error of the mean (SEM). When the two groups showed normal distribution, a two-tailed unpaired t-test, in other cases a nonparametric Mann–Whitney-U test was calculated comparing the two groups (control versus SGCE). Two-way ANOVA followed by Bonferroni post hoc analysis was used for multiple comparisons, when comparing more than two groups. The significance level (*p*-value) was set to *p* < 0.05 with * *p* < 0.05, ** *p* < 0.01, *** *p* < 0.001.

## Figures and Tables

**Table 1:** Whole-cell patch-clamp recordings of iPSC-derived MSNs from two DYT-SGCE patients and two healthy controls.

	Control			DYT-SGCE		
Functional Properties	Control_1 (*n* = 21)	Control_2 (*n* = 14)	Control (*n* = 35)	SGCE_1 (*n* = 24)	SGCE_2 (*n* = 19)	SGCE (*n* = 43)
I_Na_ max. amplitudes (pA/pF)	−106.2 ± 18.8	−126.6 ± 19.5	−116.4 ± 10.2	−85.75 ± 12.3	−92.78 ± 14.72	−89.27 ± 3.52
I_K_ max. amplitudes (pA/pF)	148.4 ± 30.2	125.3 ± 26.3	136.9 ± 11.5	103.8 ± 13.1	128.4 ± 19.3	116.1 ± 12.3
Resting membrane potential (mV)	−39.1 ± 2.2	−23.3 ± 3.1	−32.9 ± 2.2	−33.4 ± 1.8	−38.33 ± 3.0	−35.7 ± 1.7
Membrane capacitance (pF)	30.5 ± 3.9	17.8 ± 3.1	25.4 ± 2.8	27.8 ± 4.6	28.2 ± 5.1	28.0 ± 3.4
Input resistance (MOhm)	996.3 ± 275.2	553.9 ± 195.2	819.4 ± 184.2	415.1 ± 74.6	566.1 ± 82.1	481.8 ± 55.8
Cells with single evoked APs (%)	48.9 ± 24.8	87.5 ± 12.5	68.2 ± 15.1	68.6 ± 2.0	72.2 ± 14.7	70.8 ± 8.1
Cells with repetitive evoked APs (%)	51.1 ± 24.8	12.5 ± 12.5	31.8 ± 15.1	31.4 ± 2.0	22.2 ± 14.7	25.9 ± 8.4
Amplitude (mV) of evoked APs	56.9 ± 5.1	57.8 ± 5.0	57.2 ± 3.7	75.8 ± 3.4	72.5 ± 4.1	74.3 ± 2.6 ***
Duration (ms) of evoked APs	3.0 ± 0.4	3.5 ± 0.7	3.2 ± 0.4	2.4 ± 0.2	2.3 ± 0.1	2.3 ± 0.1 *
AHP amplitude (mV)	7.6± 1.9	3.2 ± 0.7	6.0 ± 1.3	8.3 ± 1.1	8.1 ± 1.4	8.2 ± 0.9 *
Time to peak AHP (ms)	21.6 ± 4.0	19.5 ± 3.0	20.9 ± 2.7	21.3 ± 2.0	31.9 ± 6.4	26.0 ± 3.1
Cells with spontaneous APs (%)	58.5 ± 20.3	37.5 ± 19.1	48.0 ± 13.3	74.5 ± 7.8	54.2 ± 23.2	62.3 ± 13.9
Frequency of spontaneous APs (Hz)	0.50 ± 0.10	0.47 ± 0.10	0.49 ± 0.07	0.52 ± 0.12	0.72 ± 0.16	0.60 ± 0.10
Amplitude of spontaneous APs (mV)	49.0 ± 3.1	42.7 ± 6.7	46.5 ± 3.2	46.7 ± 2.9	38.9 ± 3.4	43.3 ± 2.3
Cells with miniature PSCs (%)	96.3 ± 3.7	100.0 ± 0.0	98.2 ± 1.9	75.0 ± 25.0	82.8 ± 8.6	79.7 ± 9.4
Miniature PSC frequencies (Hz)	3.2 ± 0.71	2.4 ± 0.63	2.9 ± 0.48	2.0 ± 0.37	2.0 ± 0.53	2.0 ± 0.30
Miniature PSC amplitudes (pA)	22.6 ± 2.2	21.0 ± 3.1	21.9 ± 1.8	35.2 ± 2.9	20.0 ± 2.4	29.8 ± 2.4 *

Abbreviations: Voltage-gated sodium (I_Na_) and potassium (I_K_) currents, action potentials (APs), after-hyperpolarization (AHP), postsynaptic currents (PSC). Data are given as means ± SEM (* *p* < 0.05, *** *p* < 0.001, nonparametric Mann–Whitney-U test).

**Table 2:** Characteristics of healthy control subjects and DYT-SGCE myoclonus-dystonia patients as skin fibroblast donors for iPSC lines used in this study.

**Controls**				
ID code	Gender	Age at biopsy		Previously published
Control_1	F	48		Japtok et al., 2015 [57]
Control_2	M	34		Glaß et al., 2018 [58]
**DYT-SGCE Patients**				
ID code	Gender	Age at biopsy	Genotype of locus	Previously published
SGCE_1	M	39	SGCE, c.298T>G	Grütz et al., 2017 [19]
SGCE_2	F	29	SGCE, c.304C>T	Grütz et al., 2017 [19]

## Data Availability

Data is contained within the article or supplementary material.

## Supplementary Materials

The following are available online at https://www.mdpi.com/article/10.3390/ijms22073565/s1, Table S1: Quantitative real-time PCR analysis of selected and reference genes for iPSCs and MSNs after 70 days of differentiation in vitro, Table S2: Quantitative real-time PCR analysis of voltage-gated Ca^2+^-channel subunit expression in MSNs after 70 days differentiation in vitro. Table S3: Quantitative real-time PCR analysis of GABA_A_ receptor subunit expression in MSNs after 70 days of differentiation in vitro. Table S4: Quantitative real-time PCR analysis of nicotinic and muscarinic acetylcholine receptor subunit expression in MSNs after 70 days differentiation in vitro.
