# Supplementary material for: Functional and Molecular Properties of DYT-SGCE Myoclonus-Dystonia Patient-Derived Striatal Medium Spiny Neurons
Source: Int J Mol Sci. 2021 Mar 30;22(7):3565. doi: 10.3390/ijms22073565 (PMC8037318; doi:10.3390/ijms22073565)
Supplement: Supplementary file 1 [file ijms-22-03565-s001.pdf]

Supplementary Table 1. Quantitative real-time PCR analysis of selected and reference genes for iPSCs and MSNs after 70 days of differentiation *in vitro*. Primer sequences and amplification product in base pairs (bp) are given.

Targets	Primer sequence (forward; reverse)	Product (bp)
GAD67	AGATCAACAAATGCCTGGAACTGGC;<br>GAGCCACCTTGTGTAGCTTTTCCC	183
FOXP1	CCACGTGGAAGAATGCAGTGCG;<br>GCATTGAGAGGTGTGCAGTAGGC	193
CTIP2	CTCCGAGCTCAGGAAAGTGTC;<br>TCATCTTTACCTGCAATGTTCTCC	129
β-tubulin III	AGTGATGAGCATGGCATCGACCC;<br>GGCACGTACTTGTGAGAAGAGGC	110
MAP2	CAGGCAAAGGACAAAGTCTCTGACG;<br>CGCCGAGGAGGGAGAATGGAGG	92
B2M	TGCCTGCCGTGTGAACCATGT;<br>TGCGGAATCTTCAAACCTCCATGA	97
GAPDH	AGCCACATCGCTCAGACACCAT;<br>CAGGCGCCCAATACGACCAAAT	71
β-actin	CATGTACGTTGCTATCCAGGC;<br>CTCCTTAATGTCACGCACGAT	250

Supplementary Table 2. Quantitative real-time PCR analysis of voltage-gated Ca^{2+}-channel subunit expression in MSNs after 70 days differentiation *in vitro*. Primer sequences and amplification product in base pairs (bp) are given.

Voltage-gated Ca^{2+}-channel subunit	Primer sequence (forward; reverse)	Product (bp)
Ca_v 1.2 (L-type)	CATTTGACGCCTTGATTGTTGTGGG; GTATGTTCAGCTGGGTTTACCTCGG	73
Ca_v 1.3 (L-type)	CGGACCCCGTCCTCGAAGGA; CCTACGCGGATCGGGTTGGT	111
Ca_v 2.1 (P-type)	CCAGAAACTTGCCCTACAGAAAGCC; CGGGTCCATTTCGTTATACAGGGC	196
Ca_v 2.2 (N-type)	TGCTGTTCAGGAGCGCCACG; CGGTGGCATTGGCCTGCTCA;	93
Ca_v 2.3 (R-type)	GTGGCCCTGGGGTTCATCTTCCATA; CAGGATGCCACTGAGGACCACGA;	90
Ca_v 3.1 (T-type)	TCAGCCTCCCCCTGAGCGTG; TTCTGCAGGACCGCATGCCG	111
Ca_v 3.2 (T-type)	GTCACTCTGCTGCTGGATACGC; TCAGGTTGTTGTTCCTGACAAAGGC	160
Ca_v 3.3 (T-type)	ATCGACTACACCCTGTGCTTCCG; GACGTAGTCGAAGAGTTTGTGGGC	162

Supplementary Table 3. Quantitative real-time PCR analysis of $GABA_A$ receptor subunit expression in MSNs after 70 days of differentiation *in vitro*. Primer sequences and amplification product in base pairs (bp) are given [66~~64~~].

$GABA_A$ receptor subunit	Primer sequence (forward; reverse)	Product (bp)
α1	TGCAGCTTGGAGACAGGATT;	97
	TGAACCATCTTCCCCCTCTT	
α2	AGAGGATGGACTTGGGATGG;	117
	AAGATTCGGGGCATAATTGG	
α3	CACAAGTGTCGTTCTGGCTCA;	99
	TGGCACTGATACTCAAGGTGGT	
α4	TCCGGTTTTCATGCAAAGGT;	100
	CTTCATTAAGGATAAGCCAGTGGAA	
α5	GGTGTCCTTTTGGCTGAACC;	117
	GCCACTTTGGGCAGAGAGTT	
α6	TTTCCCAGGTGTCTTTCTGGA;	101
	GGCACTGATGCTCAAAGTGG	
β1	ATGCATCTGCAGCCAGAGTC;	95
	AGGGATCTTTGGCAGGGTCT	
β2	CCCAAACCAAATGTCACTGC;	90
	TGGAACTGTCAACTTGCTTCAAA	
β3	ATTGAAAGGCGCCATGTTTT;	104
	GGGTTGGTCCTAGGGAGAGG	
γ1	GGAGATGGGGGATGATAGGC;	105
	ATCCCTTCCACCCAACACAC	
γ2	TTGTCGAACAGGAGCTTGGA;	91
	GAAGGCAGTGGGGAAGAAGA	
γ3	AACCAACCACCACGAAGAAGA;	113
	CCTCATGTCCAGGAGGGAAT	
δ	GTCTTTGCTCTGCAGGATCG;	124
	CCAGGCCAAGGCTTTATTTC	

Supplementary Table 4. Quantitative real-time PCR analysis of nicotinic and muscarinic acetylcholine receptor subunit expression in MSNs after 70 days differentiation *in vitro*. Primer sequences and amplification product in base pairs (bp) are given.

Nicotinic and muscarinic acetylcholine receptor subunit	Primer sequence (forward; reverse)	Product (bp)
CHRNA3	CCTGCACAGAAGATCTGGAAGCC; CTTGTCGTCCACCTGGAAATCCC	75
CHRNA4	CAGATGATGACCACGAACGTATGGG; TGTTGTAGAGGACGATGTCCGGC	142
CHRNA5	TCTAGAAACACATTGGAAGCTGCG; GAAACATCCGATCAAGAACCTGGG	130
CHRNA6	ACACTTTGAAGTGGCCATCACCC; GGAACGCGAAGAGTCTCAATGCC	105
CHRNA7	AACCACTCACCGTCTACTTCTCCC; TGATCTGTCCAAGACATTTGCAGCC	109
CHRNB2	TACAGCTTATGGTGTCACTGGCCC; TCCAGGTGAGGCGATAATCTTCCC	111
CHRNB4	GGACGACCTTCTGAACAAAACCCG; CTCATTCACGCTGATAAGCTGGGC	118
CHRM1	TCAAGAGGCCGACTAAGAAAGGGC; CACCAGCACCATGATGTTGTACGG	185
CHRM2	AAATGAATCCAGCCCAGCTCGC; AGGAGTCCTTTGTGGGTTCAGGG	173
CHRM3	ACGATCTTAAGGACAGTCGCTCCC; ACACCTAAGTTCAGATCCTGGCCC	176
CHRM4	CTACTTAGCCAGGTTCTGGGTGGG; TCTGAATACGTGGACCACTCACGG	174
CHRM5	TACTGAGCTTCAAACAAACCACTGCC; GGTGGTTGCATTGTGGTAAGAATCCC	136
